# Synergistic Inhibition of Drug Resistant KRAS Mutant Non-Small Cell Lung Cancer by Co-Targeting AXL and SRC

**DOI:** 10.3390/cancers17030490

**Published:** 2025-02-01

**Authors:** Soumavo Mukherjee, Dhananjay Suresh, Ajit Zambre, Sairam Yadavilli, Shreya Ghoshdastidar, Anandhi Upendran, Raghuraman Kannan

**Affiliations:** 1 Department of Bioengineering, University of Missouri, Columbia, MO 65211, USA; 2 Department of Radiology, University of Missouri, Columbia, MO 65212, USA; 3 Department of Medical Pharmacology & Physiology, University of Missouri, Columbia, MO 65212, USA; 4 Ellis Fischel Cancer Center, University of Missouri, Columbia, MO 65212, USA

**Keywords:** AXL, Dasatinib, drug resistance, KRAS, SGI-7079, SRC, TKI

## Abstract

Background/Objectives: KRAS-mutated NSCLC has been targeted using monoclonal antibody (mAb) or tyrosine kinase inhibitor (TKI) therapies. However, in time, these mutations appear to develop resistance against the targeted antibodies and TKI treatments. One possible explanation is the activation of pro apoptotic pathways through the AXL–SRC–Akt axis. In this study, we identify AXL as the bypass resistant gene and investigate its role with KRAS and SRC activity. Methods: In this study, we use Dasatinib and SGI-7079 to co-inhibit SRC and AXL respectively. In vitro studies were conducted using four cell lines, and AXL suppression was achieved using siRNA and in CRISPR-Cas9 mediated knockout models. Subsequently, we studied gene-protein expression analysis using Western blot, apoptotic markers using a cytochrome release assay and cytotoxicity using an MTT assay. A549 xenografts were studied for in vivo validation of our proposed hypothesis. Results: The results suggest that dual inhibition of AXL and SRC significantly reversed this resistance, both in in vivo and in vitro studies. Conclusions: Co-inhibition of AXL and SRC synergistically reduced KRAS activity and induced apoptosis in NSCLC.

## Simple Summary

Non-small cell lung cancer (NSCLC) patients harboring KRAS mutations are targeted using monoclonal antibody (mAb) or tyrosine kinase inhibitors (TKI) therapies. To impede the KRAS activity, it was proposed to inhibit SRC kinase, an intermediary signal transductor between DDR2 and KRAS, using Dasatinib (Bcr-Abl TKI). However, clinical trials using Dasatinib failed to fully inhibit SRC or sensitize therapies towards SRC inhibitors, and its mechanistic failure remains partially understood. This report identifies AXL as a bypass resistant gene and investigates its role with SRC and KRAS activity. We found that AXL overexpression drives downstream KRAS and Akt pathways, and co-inhibiting AXL and SRC using SGI-7079 and Dasatinib effectively impeded both those pathways. Synergistic inhibition of AXL and SRC led to significant tumor growth suppression in A549 mice xenografts. The results from this study will be beneficial in understanding drug-resistance mechanisms and to strategize effective drug therapies in patients harboring KRAS mutations.

## 1. Introduction

Non-small cell lung cancer (NSCLC) remains a high-mortality disease as patients often develop resistance to treatment. Patients with mutations in EGFR or KRAS initially respond well to small-molecule inhibitors but quickly stop responding to the drug. Different mechanisms have been proposed to predict drug resistance, among which AXL is well-known and is overexpressed in patients with EGFR mutations and KRAS mutations [1,2]. Small-molecule inhibitors targeting AXL are being developed to overcome drug resistance. These inhibitors are most effective when used as a combination therapy rather than a standalone treatment. Various combinations have been identified and are currently being evaluated in preclinical or clinical trials, and the most efficacious combination is yet to be identified [3]. To determine the right combination therapy for controlling NSCLC tumors, we assessed the effectiveness of combining AXL and SRC inhibitors in regulating tumor growth using human cell lines and an animal CDX model.

AXL is a member of the TAM family, which also includes TYRO3 and MERTK. It is a transmembrane receptor tyrosine kinase. The extracellular domain of AXL contains a binding site for the growth arrest specific-6 (GAS-6) ligand. When GAS-6 binds to AXL, receptor dimerization occurs, activating the intracellular kinase domain. This activation triggers several downstream signaling pathways, including the PI3K/AKT, MAPK, and NF-κB pathways. These pathways contribute to cell survival, proliferation, migration and invasion [4]. The upregulation of AXL is an adaptive response to treatment pressures, especially observed during the treatment with EGFR or KRAS inhibitors. AXL overexpression in cancer is associated with aggressive disease progression and often associated with poorer clinical outcomes in NSCLC patients. Based on these robust data, AXL is emerging as a therapeutic target, and several small molecule inhibitors are being developed and evaluated for combination therapy. The ongoing research and development of AXL inhibitors, such as Bemcentinib, Cabozantinib, Gilteritinib, and TP-0903, hold promise for improving treatment outcomes in drug-resistant NSCLC patients. Targeting AXL may offer new avenues for combination therapies, and the studies highlight the need for continued exploration to ultimately overcome drug resistance in NSCLC [4].

SRC is an important target in drug-resistant NSCLC. SRC is overexpressed in NSCLC and promotes tumor growth and proliferation. Lung tissue sample data from patients confirm the activation of SRC-family kinases, which have been identified as a key mechanism behind drug resistance. For instance, SRC regulates YAP, a known promoter of drug resistance and cancer progression in NSCLC [5,6,7]. The interaction between SRC and other pathways suggests the potential for combination therapies. Although pre-clinical studies have indicated that Dasatinib, an SRC inhibitor, shows promise for treating NSCLC subjects [8], clinical trials showed modest response when compared to established chemotherapeutic agents [9]. Notably, dual inhibition of both SRC and DDR2 leads to enhanced suppression of cancer growth [10,11]. These findings have prompted us to explore the combination of AXL and SRC targeting in controlling tumor growth.

In this study, we want to investigate whether combining AXL and SRC inhibitors effectively controls NSCLC tumors. The rationale for the dual inhibition in drug-resistant NSCLC is based on their complementary roles in resistance mechanisms and tumor progression [12]. As previously mentioned, AXL is a mediator for resistance, while SRC is a central node in several signaling pathways that enhance cancer cell survival and metastasis. Both kinases can independently activate overlapping downstream pathways, such as the PI3K/AKT and MAPK signaling pathways. These data suggest that dual inhibition may provide a comprehensive blockade of these pathways and reduce the possibility of compensatory activation. Simultaneous inhibition of both AXL and SRC could prevent resistance and control tumor progression. We examined the effect of AXL inhibition, alone and in combination with SRC inhibition, in KRAS mutant NSCLC mouse model. Our studies confirmed that dual inhibition of AXL and SRC led to enhanced cell death and reduced tumor growth in both in vitro and in vivo models.

## 2. Materials and Methods

### 2.1. Materials, Instrumentation and Software

Dasatinib and AXL inhibitor, SGI-7079, were purchased from Selleck chemicals (Houston, TX, USA). Tween-20, bovine serum albumin (BSA), MS-SAFE Protease and Phosphatase Inhibitor, triton X-100, phosphate buffer saline (PBS), cell dissociation buffer and cell grade DMSO were purchased from Sigma-Aldrich. 2-mercaptoethanol, Pierce ECL Western Blotting Substrate, Pierce BCA Protein Assay Kit and UltraPure DNase/RNase-Free Distilled Water were purchased from Thermo-Fisher Scientific (Waltham, MA, USA). Edit-R predesigned CRISPR kit and sulfur-group-linked siRNA were custom synthesized from Horizon Discovery (Waterbeach, UK). TransIT-X2 transfection agent was purchased from Mirus Bio LLC (Madison, WI, USA). Furthermore, 10× TBS, 10× Tris/Glycine/SDS buffer, 4–15% 10-well 50 µL ready Mini-Protean TGX gels, Precision Plus Protein Dual color standard ladder and supported nitrocellulose Membrane (0.2 µm) were purchased from Bio-Rad (Hercules, CA, USA). Primary antibodies against DDR2, AXL, p-SRC (T416), cleaved-PARP, phospho-SHP2 (T542), phospho-SHP2 (T580), Survivin, Actin, cleaved-Caspase 3, and HRP-conjugated anti-rabbit, Alexa-fluor^®^ 647 attached secondary antibody and anti-mouse IgG antibodies were purchased from Cell Signaling (Danvers, MA, USA). Phospho-DDR2 was purchased from R&D Systems (Minneapolis, MN, USA). Athymic nude mice were purchased from Charles River Laboratories International Inc. (Wilmington, DE, USA).

Gel electrophoresis was performed on a Bio-Rad Mini-Protean Tetra system and blots were transferred using a Biorad Transblot Turbo transfer system. Western blot image acquisition was performed using Image Lab 5.2.1 software on a ChemiDoc XRS system from Bio-Rad, and densitometry was performed using Image Studio Lite (Li-COR Biotechnology, Lincoln, NE, USA). The spectral absorbance was measured at 570 nm to 620 nm using the Cytation 5 microplate reader. The results were analyzed using Biotek Gen5 image+ program. The slides were imaged using a Leica (DM5550 B) microscope. Gene expression analysis was performed on a QuantStudio™ 3 Real-Time PCR System (Applied Biosystems™, Waltham, MA, USA). The cells were analyzed using BD CyAn ADP high performance flow cytometer, and histograms were generated using FlowJo 10 (FlowJo, Ashland, OR, USA). Statistical analysis was performed on GraphPad Prism 7 software (Boston, MA, USA).

### 2.2. Inhibitor Reagent Preparation

All were suspended in cell-grade DMSO. A Dasatinib concentration of 40 µM was used (used within 24 h of preparation). In a similar fashion, the AXL inhibitor was dissolved in cell-grade DMSO to obtain 0.1 µM concentration (used within 24 h of preparation). The solutions were stored at −4 °C.

### 2.3. Cell Lines and Knockout Model Generation

All NSCLC cell lines A549, H460, H2122, and H441 were purchased from ATCC (American Type Culture Collection; Manassas, VA, USA) and cultured as described by the ATCC. The cell lines were maintained at 37 °C with 5% CO_2_ in 10% RPMI medium 1640 by ATCC. To develop A549 AXL knockout cells we targeted site Exon 9 (NM_001278599) of the human AXL gene, using 200 µL of crRNA complex (Edit-R predesigned crRNA) containing transfer RNA, CRISPR-Cas9 mRNA and transfection agent (dissolved in serum free media, and incubated for 72 h), followed by single cell purification and isolation. The final knockout cells were tested by Western blot, tested for mycoplasma and stored in liquid nitrogen.

### 2.4. RNAi

Small interfering RNA (siRNA) for AXL (sense: S-SGGAACUGCAUGCUGAAUGAUU, antisense: UCAUUCAGCAUGCAGUUCCUU) were used. For our experiments, 1 × 10^6^ cells per mL were incubated overnight in a custom RPMI medium, enriched with 10% FBS. The cells were then transfected with siRNA using the TransIT-X2 transfection agent and incubated overnight in serum-free media before being treated for Western blot. For toxicity study and immunocytochemistry, 2 × 10^5^ cells/mL and 5 × 10^5^ cells/mL were seeded using the same above protocol, respectively.

### 2.5. Western Blotting

To prepare the cell lysates, 1 × 10^6^ cells/mL were seeded overnight in a custom RPMI medium, enriched with 10% FBS. Then the cells were washed with PBS and incubated for 48 h, followed by treatment with Dasatinib (40% of IC_50_ value of each cell line) and 0.1 µM of AXL inhibitor. The cells were lysed using MS-SAFE protease and a phosphatase inhibitor cocktail and protein estimations were performed using a bicinchoninic acid (BCA) assay. The samples were subjected to PAGE (polyacrylamide gel electrophoresis) using 4–15% 10-well 50 µL ready Mini-Protean gels and blotted onto a nitrocellulose membrane (0.2 um), immunostained and imaged for chemiluminescence.

### 2.6. MTT Assay

Cell proliferation was determined using a 3-(4, 5-dimethylthiazol-2-yl)-2, 5-diphenyltetrazolium bromide (MTT) assay that detects the cellular capacity to convert tetrazolium salt to formazan. Briefly, 2 × 10^6^ cells/mL of each cell line were plated in a 96-well plate. After 24 h incubation in FBS enriched RPMI, the medium was removed and replaced with 0.1 µM AXL inhibitor/Dasatinib of decreasing concentration (50% of each previous dose), starting from 40 µM in serum free media, by serial dilution. After 48 h of treatment, 10 µL of MTT dye was added, followed by the addition of stop solution after 6 h, as per manufacturer’s protocol. The plates were read for absorbance at 570 nm.

### 2.7. Cytochrome-C Release Assay

First, 5 × 10^5^ cells/mL A549 cells were seeded on to a coverslip, pre-coated with poly-L-lysine for 24 h. The cells were then treated with 40% IC_50_ value of Dasatinib of the respective cell line and 50 µM of AXL-siRNA. The cells were fixed using 4% paraformaldehyde in PBS for 20 min in room temperature. To improve the signal, the cells were treated at 95 °C with antigen retrieval buffer (100 mM Tris, 5% urea (*w*/*v*), pH 9.5). The cells were made permeable using 0.1% Triton-X 100 for 10 min at room temperature, followed by blocking with 10% goat serum for 1 h at room temperature and incubation with primary antibodies, diluted in 10% goat serum for 2 h at room temperature. The cells were then rinsed with 1% goat serum, away from light, and again incubated for 1 h at room temperature with fluorophore-conjugated secondary antibodies, diluted in 10% goat serum. The coverslips were rinsed again in 1% goat serum, and DAPI was added at the final wash to stain the nuclei. The manufacturer’s protocol was followed [ApoTrack™ Cytochrome-c Apoptosis ICC Antibody Kit (ab110417)], and the slides were imaged using fluorescence microscopy.

### 2.8. Flow Cytometry

First, 2 × 10^6^ cells/mL of A549 cell line were treated with either AXL or SRC inhibitors or a combination of both (48 h) and then harvested using PBS-based cell dissociation buffer. Next, cells were fixed using BD Cytofix/Cytoperm™ solutions that contain 4% paraformaldehyde. The fixed cells were then incubated for 1.5 h with AXL or SHP2 primary antibodies, followed by an Alexa-fluor^®^ 647 attached secondary antibody incubation for 30 min. Three washes using saponin-based wash buffer were performed after fixing and after each incubation step. The cells were analyzed with a high-performance flow cytometer.

### 2.9. Animal Studies

First, 1 × 10^7^ cells/mL of A549 cells were injected at the left flank to stimulate tumor growth. Mice were distributed in 6 groups of 8 animals each (4 males and 4 females). SRC inhibitor Dasatinib and AXL inhibitor SGI-7079 were administered 5 days a week orally at 40 mg/kg and 25 mg/kg, respectively. All orally administered solutions were dissolved in DMSO to reach their desired concentration and then in propylene glycol and water at 1:1 ratio to reach the final administration volume of 200 µL per animal per dose. Both oral and IP vehicle were administered to the control group in the same way as the treatment mice. Tumor sizes were monitored twice weekly, and volumes were calculated with the formula: (mm^3^) = length × width × width × 0.5. Animals were sacrificed as per IBC protocol and regulations.

### 2.10. Statistics

All tests were averaged from triplicate. For the MTT assay, cell-free duplicate columns were used, blanking each concentration dose. Two-way ANOVA and Fisher’s LSD test or ordinary one-way ANOVA and Tukey’s multiple comparison test, with a single pooled variance, were performed. A *p*-value less than 0.05 was considered statistically significant and was represented in figures as * (*p* < 0.05), ** (*p* < 0.01), *** (*p* < 0.001) or **** (*p* < 0.0001). Mean ± SEM was represented for all experiments.

## 3. Results

### 3.1. Co-Inhibition of AXL and SRC Synergistically Reduces KRAS Activity and Induces Apoptosis in Cancer Cell Lines

**Figure 1:**
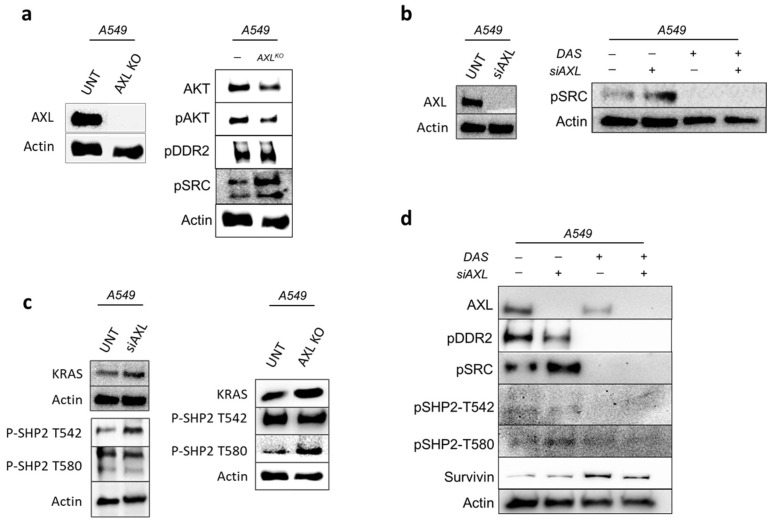
Downstream effects of AXL and SRC in KRAS mutant NSCLC. (**a**) Western blot image showing protein expression of AXL- and SRC-related markers in AXL knockout A549 cells. (**b**) Western blot image showing protein expression for AXL and phospho-SRC in A549 cells treated with either siAXL, Dasatinib or both. (**c**) Western blot showing protein expression of KRAS and phospho-SHP2 (T542 and T580) in A549 cells before or after siAXL or knockout treatments. Increased expression in SHP2 activity is observed when AXL is inhibited. (**d**) Western blot image for downstream KRAS activity in A549 cells treated with either siAXL, Dasatinib or both.

Our previous research and other independent studies have shown that AXL overexpression can regulate the PI3K/Akt pathway and activate SRC [3,12,13]. This result led us to explore the effects of inhibiting both AXL and SRC. For our study, we selected two cell lines from the available options—A549 with KRAS-G12S mutation and H460 with KRAS-Q61H mutation. For the combined inhibition of AXL and SRC, we employed siRNA targeting AXL and Dasatinib to inhibit SRC (Dasatinib is an FDA-approved SRC inhibitor). Previous studies have indicated that AXL is associated with DDR2, a SRC phosphorylator [14]. Interestingly, SRC can also phosphorylate DDR2 in a reinforcing-feedback loop [15,16]. Therefore, the DDR2 levels measured before or after inhibition provide critical information about efficacy. Our studies used Western blot to monitor the change in AXL, phospho-SRC, phospho-DDR2, and Akt protein levels in the wild type and AXL-knockout cell lines (Figure 1a). When AXL is knocked out or downregulated in A549 cells, the levels of phosphorylated SRC and DDR2 increase, while Akt activity decreases (Figure 1a,b). Inhibition of AXL alone resulted in the autophosphorylation of SRC when DDR2 was present and thus may lead to uninhibited PI3K-Akt and KRAS downstream activity (Figure 1c) [17]. So, we co-inhibited AXL and SRC and found that phosphorylated SRC homology region 2-containing protein tyrosine phosphatase 2 (SHP2) and DDR2 levels decreased (Figure 1d and Figure S1). We assessed the protein expression levels of SHP2 at tyrosine residues 542 and 580, which are known to activate RAS and are also associated with DDR2 downstream signaling [18]. Survivin, an inhibitor of apoptosis (AIP) family protein increased when cells were treated with Dasatinib; however, protein expression decreased during co-inhibition, indicating reduced cell compensatory activity (Figure 1d). DDR2, SHP2, and Survivin activity reduction indicate pro-apoptotic cell activity.

**Figure 2:**
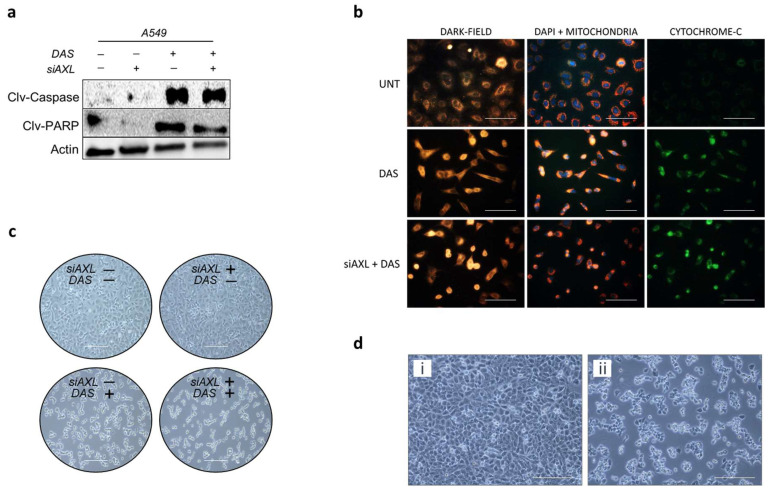
Effect of co-inhibiting AXL and SRC in KRAS mutant NSCLC. (**a**) Western blot image for apoptotic proteins in A549 cells treated with either siAXL, Dasatinib or both. (**b**) Cytochrome-C assay for A549 cells; scale bar = 100 µm. (**c**) Cell-Death assay: Bright field Microscopy images (20×) for triggered cell death in A549 cells before or after either siAXL or DAS or treatment with both; scale bar = 200 µm. (**d**) Cell-Death assay: Bright field microscopy images (20×) of H460AK cells (i) before and (ii) after treatment with Dasatinib (DAS) for 48 h; scale bar = 200 µm. Results indicate higher cell death after DAS treatment in AXL knockout H460 cells.

To further verify, we estimated the expression levels of pro-apoptotic proteins such as cleaved Caspase, cleaved PARP, and assessed cytochrome-C leakage from the mitochondrial wall to cytosol. Co-inhibition of AXL and SRC resulted in increased expression of cleaved Caspase and cleaved PARP, indicating a greater likelihood of cell death (Figure 2a). After staining the mitochondrial membrane with a mitochondrial marker and cytochrome-C in the cytosol using fluorophore-conjugated monoclonal antibodies, the dual-inhibited cells A549 exhibited a significantly greater green fluorescence signal (indicating cytochrome-C) in the cytosol surrounding the mitochondria (shown by the red signal) compared to the cells treated with Dasatinib alone (see Figure 2b and Figure S2). This finding supports the higher expression of pro-apoptotic proteins. We observe increased cell death under dual-inhibition conditions compared to inhibiting AXL or Dasatinib alone (Figure 2c) in the cell death assay using microscopy. Significant cell death was observed in AXL CRISPR-knockout cells treated with Dasatinib (Figure 2d).

**Figure 3:**
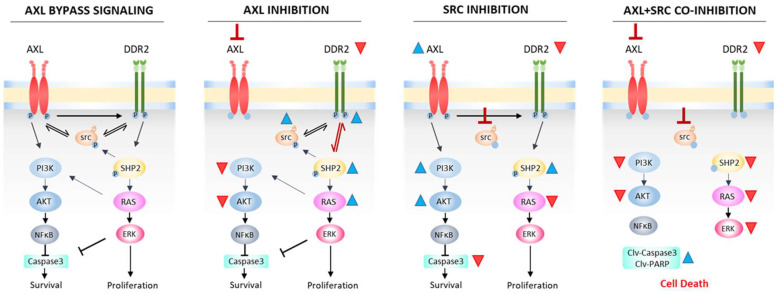
Postulated molecular mechanism for AXL bypass signaling and therapy.

These results align with both the Western blot and cytochrome-C release assays. Together, the data show that co-inhibition of AXL and SRC effectively blocked KRAS activity and promoted cell death (Figure 3).

### 3.2. Synergistic Effects of AXL and SRC: In Vitro Study

**Figure 4:**
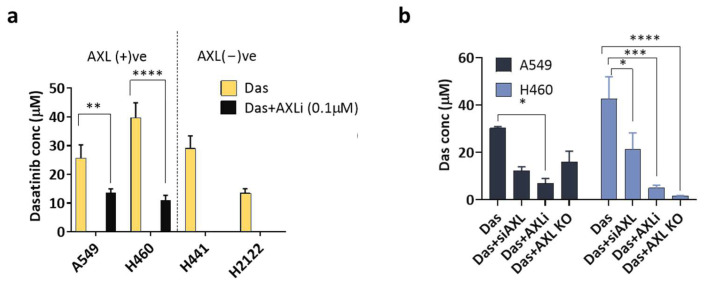
Synergistic AXL and SRC therapy in vitro. (**a**) Dasatinib (IC_50_) sensitization of NSCLC cell lines after co-inhibition of AXL + SRC using MTT assay (72 h). (**b**) Dasatinib (IC_50_) sensitization of A549 and H460 cell lines comparing co-inhibition of AXL + SRC using siAXL or AXLi or CRISPR-knockout methods in an MTT assay (72 h). *p*-values are denoted by * (*p* < 0.05), ** (*p* < 0.01), *** (*p* < 0.001) or **** (*p* < 0.0001).

Inhibition of AXL and SRC led to increased levels of pro-apoptotic proteins in cancer cells. This finding prompted us to explore the potential of dual inhibition as a combined therapy for controlling cancer cell proliferation. To investigate this approach further, we conducted experiments using in vitro cancer cells and in vivo mice models. Our study focused on two groups of cell lines: those expressing AXL (AXL +ve) and those lacking AXL expression, referred to as AXL-negative. We selected AXL +ve A549 and H460 cells and AXL −ve H441 and H2122 cells for our investigation. We employed SGI-7079 and Dasatinib as inhibitors for AXL and SRC, respectively. In our initial experiment, we examined the effectiveness of simultaneously inhibiting both AXL and SRC in various cancer cell lines (Figure 4a). We hypothesized that inhibiting AXL would enhance cellular sensitivity to SRC, leading to a lower IC_50_ value for Dasatinib than cells lacking AXL inhibition. We applied a consistent concentration of an AXL inhibitor (0.1 µM) while systematically varying the concentration of Dasatinib to determine the IC_50_ values. In the cell lines that express AXL, we observed a significant decrease in the IC_50_ values following AXL inhibition, resulting in a dose reduction of 66% to 93% (18 µM to 6 µM in A549 and 39 µM to 2.5 µM in H460). In contrast, AXL-negative cells did not show any change in IC_50_ values; these results are anticipated because AXL is not a primary driver of growth in these cell lines. As a next step, we aimed to investigate whether the effectiveness of SRC inhibition is contingent upon the method employed for AXL inhibition, specifically examining the use of siRNA, AXLi, and CRISPR-Knockout techniques. We observed a slight variation in the IC_50_ values; nonetheless, AXLi demonstrated a minor advantage compared to pure inhibition, likely attributable to its pleiotropic effect (Figure 4b). According to this information, we can verify that the effectiveness of SRC is minimally influenced by the method of AXL inhibition.

### 3.3. Synergistic Effects of AXL and SRC: In Vivo Study

**Figure 5:**
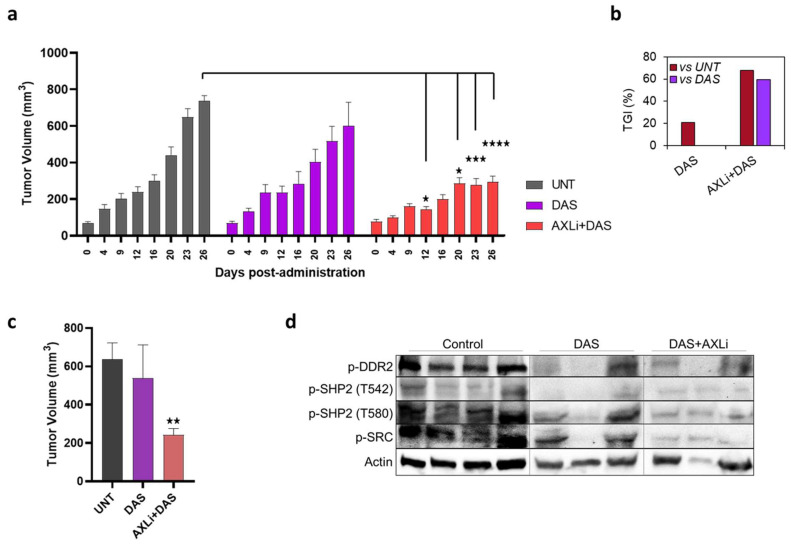
Synergistic AXL and SRC therapy in vivo. (**a**) Tumor growth reduction in A549 xenografts treated with Dasatinib or combination of AXLi and Dasatinib (**b**) Corresponding graph showing tumor growth inhibition (%). (**c**) Tumor volume reduction plot for excised tumors. (**d**) Western blot image for DDR2, SHP2 and SRC activity in tumor lysates at the end of study. *p*-values are denoted by * (*p* < 0.05), ** (*p* < 0.01), *** (*p* < 0.001) or **** (*p* < 0.0001).

To evaluate the therapeutic effectiveness of combined SRC and AXL inhibitors in vivo, we selected the A549 CDX model due to its overexpression of AXL and SRC. The study included three groups: untreated, treated with Dasatinib alone and treated with the combination of Dasatinib and AXL inhibitors. Treatment began after 12 days of tumor growth, following baseline measurements. We hypothesized that the combination treatment would more effectively control tumor growth than the other two groups. Since AXL inhibitors alone have not demonstrated significant benefits in cell studies, we did not include a separate group for that treatment. The objective of this study is to enhance the efficacy of Dasatinib when used in combination with AXL inhibitors. As illustrated in Figure 5a,b, there was no notable change in tumor growth for the Dasatinib group compared to the untreated group. In contrast, a significant and rapid reduction in tumor growth was noted in the AXLi + Dasatinib treated group starting from the 12th day of measurements (Figure 5a). The dual inhibitor group demonstrated an average reduction of approximately 60% (*p* < 0.001) in tumor volume in comparison to the untreated group. While the Dasatinib group exhibited a tumor growth inhibition of around 20% compared to the untreated group, the dual inhibitor group achieved up to 70% inhibition relative to the untreated group (Figure 5b). Moreover, the dual inhibitor group displayed a tumor growth inhibition of approximately 60% relative to the Dasatinib-treated group (Figure 5b). We also assessed the tumor size after excising the tumors, and the results indicated a reduction in tumor volume of up to 60% (*p* < 0.1) for the dual inhibitor group when compared to the untreated group (Figure 5c). No significant changes were observed for the Dasatinib-treated group. Additionally, we analyzed the DDR2, SHP2 and SRC activity levels in the tumor lysates using Western blotting (Figure 5d), observing a significant decrease in protein levels for the dual inhibitor group relative to the untreated group. These findings suggest a successful inhibition of KRAS activity downstream of SRC following the dual inhibitor (AXLi + Dasatinib) therapy, contributing to tumor sensitization and the subsequent reduction in tumor volume.

## 4. Discussion

KRAS mutant NSCLC poses a therapeutic challenge due to its reliance on adaptive survival mechanisms, which enable resistance to targeted therapies [19]. The previous studies have shown that Dasatinib, a SRC inhibitor, achieves limited efficacy due to the activation of compensatory pathways [9], particularly those mediated by RTKs such as AXL [20]. AXL, a member of the TAM family of RTKs, is implicated in activating survival pathways, including RAS/RAF/MAPK and PI3K/Akt/mTOR, which are critical for tumor proliferation and resistance [4]. Notably, inhibition of SRC often triggers upregulation of RTKs as a feedback mechanism to sustain oncogenic signaling, thereby reducing the therapeutic efficacy of SRC-targeted treatments [20]. Based on these findings, we hypothesized that simultaneous inhibition of AXL and SRC could effectively suppress this feedback loop, reduce KRAS-driven signaling and enhance the therapeutic potential of Dasatinib in KRAS mutant NSCLC. Our findings strongly support the hypothesis that AXL plays a role in resistance to Dasatinib in KRAS mutant NSCLC. Using both pharmacological inhibition with SGI-7079 and genetic suppression with siRNA, we demonstrated that AXL inhibition significantly sensitizes KRAS mutant NSCLC cell lines, such as A549 (KRAS G12S) and H460 (KRAS Q61H), to Dasatinib. Notably, A549 and H460 cells exhibited a reduction in Dasatinib IC_50_ values by three-fold and sixteen-fold, respectively. In contrast, the AXL-negative H441 cell line (KRAS G12V) showed no significant change in IC_50_ values, indicating the AXL’s role in resistance. These results indicate that the suppression of AXL disrupts key survival pathways, enhancing the cytotoxic effects of Dasatinib. Furthermore, we observed that dual inhibition of AXL and SRC disrupted DDR2 and downstream KRAS signaling. DDR2, a key mediator in resistance mechanisms, was partially downregulated upon AXL suppression. The combination of Dasatinib and SGI-7079 further abrogated downstream signaling through RAS and its effectors, significantly increasing pro-apoptotic markers such as cleaved caspase-3 and cleaved PARP. Additionally, immunocytochemistry revealed increased cytosolic cytochrome-C levels, confirming the activation of mitochondrial apoptosis pathways. In vivo studies using A549 xenografts corroborated these findings. Dual inhibition of AXL and SRC produced superior tumor growth inhibition (TGI ~70%) compared to either monotherapy. This robust therapeutic response highlights the necessity of targeting AXL as a critical node in overcoming adaptive resistance to Dasatinib. This study demonstrates the therapeutic potential of dual inhibition of AXL and SRC in addressing resistance mechanisms in KRAS mutant NSCLC. Our findings reveal that AXL suppression disrupts compensatory feedback loops that maintain KRAS-driven oncogenic signaling during SRC inhibition, thereby enhancing the efficacy of Dasatinib. Dual inhibition impaired DDR2 and KRAS activity and induced robust apoptotic responses through mitochondrial pathways, leading to significant tumor growth inhibition in vivo. This study lays the fundamental groundwork for developing more effective combination therapies in this challenging subset of lung cancer by elucidating the molecular mechanisms underlying these synergistic effects. Further studies are warranted to understand the detailed mechanism and unequivocal confirmation of therapeutic efficacy, as our studies are limited to one cell line. However, our study highlights the vital role of AXL in adaptive resistance, supporting clinical strategies that target both AXL and SRC to enhance outcomes in KRAS mutant NSCLC.

## 5. Conclusions

In this study we demonstrate the therapeutic potential of synergistic treatments in KRAS mutant NSCLC targeting AXL and SRC. Our findings indicate that targeting SRC alone in NSCLC may activate an AXL RTK switch that will impede complete shutdown of KRAS activity downstream. We show that dual inhibition of AXL and SRC led to impairment of DDR2 regulation and downstream KRAS activity. Furthermore, we show that dual inhibition leads to successful activation of pro-apoptotic markers in the cell, release of cytochrome-C in the cytosol, cell-death and subsequent sensitization of cells towards Dasatinib. Finally, we validated that co-inhibition of AXL and SRC significantly reduced tumor growth in A549 xenografts. In conclusion, the results from this study support the therapeutic potential of dual inhibition of AXL and SRC towards KRAS mutant NSCLC therapies and will aid in our understanding of molecular mechanisms behind TKI therapies in KRAS mutant harboring cancers.

## Data Availability

All data related to this publication is presented in the main text or Supporting Information.

## Supplementary Materials

The following supporting information can be downloaded at: https://www.mdpi.com/article/10.3390/cancers17030490/s1, Figure S1: Flow cytometry data for AXL and SHP2 (inactivated) expression in A549 cells before and after treatment with Dasatinib (DAS), siAXL or a combination of both for 48 h. Results indicate a spike in AXL expression when SRC is inactivated and a spike in inactivated SHP2 when both AXL and SRC are co-inhibited; Figure S2: Quantification of cytochrome-C fluorescence intensity in A549 cells before and after treatment with DAS or siAXL + DAS.
