# Supplementary material for: Synergistic Inhibition of Drug Resistant KRAS Mutant Non-Small Cell Lung Cancer by Co-Targeting AXL and SRC
Source: Cancers (Basel). 2025 Feb 1;17(3):490. doi: 10.3390/cancers17030490 (PMC11816174; doi:10.3390/cancers17030490)
Supplement: Supplementary file 1 [file cancers-17-00490-s001.zip › cancers-3398796-supplementary.pdf]

SUPPORTING INFORMATION

Synergistic Inhibition of Drug Resistant KRAS Mutant Non-Small Cell Lung Cancer by Co-Targeting AXL and SRC

Soumavo Mukherjee [1], Dhananjay Suresh [1], Ajit Zambre [2], Sairam Yadavilli [1], Shreya Ghoshdastidar [1], Anandhi Upendran [3] and Raghuraman Kannan [1,2,4,*]

1 Department of Bioengineering, University of Missouri, Columbia, MO 65211, USA
2 Department of Radiology, University of Missouri, Columbia, MO 65212, USA
3 Department of Medical Pharmacology & Physiology, University of Missouri, Columbia, MO 65212, USA
4 Ellis Fischel Cancer Center, University of Missouri, Columbia, MO 65212, USA
* Correspondence: kannanr@health.missouri.edu

SUPPORTING FIGURES

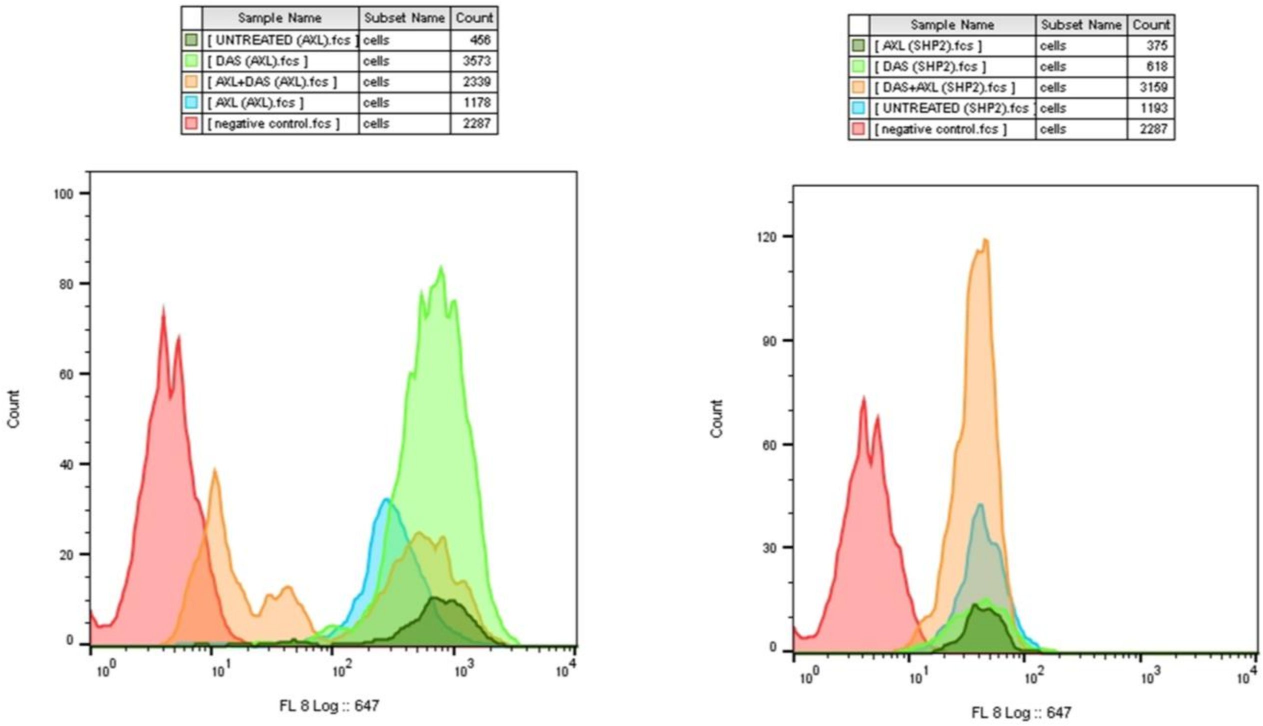

Figure S1. *Flow Cytometry*. Flow cytometry data for AXL and SHP2 (inactivated) expression in A549 cells before and after treatment with Dasatinib (DAS), or siAXL, or combination of both for 48 h. Results indicate a spike in AXL expression when SRC is inactivated and a spike in inactivated SHP2 when both AXL and SRC are co-inhibited.

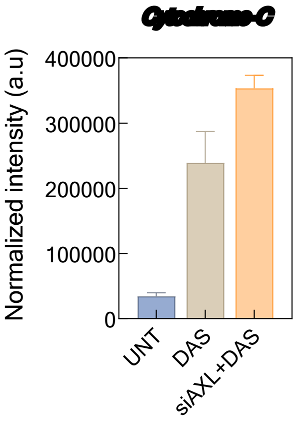

Figure S2. *Fluorescence analysis*. Quantification of cytochrome-C fluorescence intensity in A549 cells before and after treatment with DAS or siAXL+DAS.
